# Medication Adherence Prediction Through Online Social Forums: A Case Study of Fibromyalgia

**DOI:** 10.2196/12561

**Published:** 2019-04-04

**Authors:** Kyle Haas, Zina Ben Miled, Malika Mahoui

**Affiliations:** 1 Department of Electrical and Computer Engineering School of Engineering and Technology Indiana University Purdue University Indianapolis, IN United States; 2 Eli Lilly and Company Indianapolis, IN United States

**Keywords:** medication adherence, fibromyalgia, Medical Expenditure Panel Survey, social forum, random forest, transfer learning

## Abstract

**Background:**

Medication nonadherence can compound into severe medical problems for patients. Identifying patients who are likely to become nonadherent may help reduce these problems. Data-driven machine learning models can predict medication adherence by using selected indicators from patients’ past health records. Sources of data for these models traditionally fall under two main categories: (1) proprietary data from insurance claims, pharmacy prescriptions, or electronic medical records and (2) survey data collected from representative groups of patients. Models developed using these data sources often are limited because they are proprietary, subject to high cost, have limited scalability, or lack timely accessibility. These limitations suggest that social health forums might be an alternate source of data for adherence prediction. Indeed, these data are accessible, affordable, timely, and available at scale. However, they can be inaccurate.

**Objective:**

This paper proposes a medication adherence machine learning model for fibromyalgia therapies that can mitigate the inaccuracy of social health forum data.

**Methods:**

Transfer learning is a machine learning technique that allows knowledge acquired from one dataset to be transferred to another dataset. In this study, predictive adherence models for the target disease were first developed by using accurate but limited survey data. These models were then used to predict medication adherence from health social forum data. Random forest, an ensemble machine learning technique, was used to develop the predictive models. This transfer learning methodology is demonstrated in this study by examining data from the Medical Expenditure Panel Survey and the PatientsLikeMe social health forum.

**Results:**

When the models are carefully designed, less than a 5% difference in accuracy is observed between the Medical Expenditure Panel Survey and the PatientsLikeMe medication adherence predictions for fibromyalgia treatments. This design must take into consideration the mapping between the predictors and the outcomes in the two datasets.

**Conclusions:**

This study exemplifies the potential and limitations of transfer learning in medication adherence–predictive models based on survey data and social health forum data. The proposed approach can make timely medication adherence monitoring cost-effective and widely accessible. Additional investigation is needed to improve the robustness of the approach and extend its applicability to other therapies and other sources of data.

## Introduction

Medication nonadherence is one of the most expensive medical expenditures. As of 2015, the cost of patient nonadherence in the United States reached US $290 billion [1]. The majority of the cost of nonadherence arises from prescriptions that are either never filled or medications that are not taken as prescribed [2]. Although the financial losses are staggering, the most prominent motivation for better adherence is saving patients whose conditions worsen due to poor compliance. Indeed, close to 125,000 deaths related to inadequate adherence were reported in the United States [3].

Identifying patients at risk and the reasons for medication nonadherence can help guide the development of remedial and preventive plans. For many years, researchers have stipulated that several factors can influence nonadherence including poor patient-doctor interactions and a lack of overall health understanding [3]. On one hand, the multitude of factors and their potential interdependence make profiling patients at risk of nonadherence difficult [3,4]. On the other hand, the digitization efforts in the health sector over the past decade have resulted in the availability of various data sources that can support the design of medication adherence–prediction models. Examples of such sources include reimbursement claims data from insurance companies, dispensed medication data from pharmacies, and medication prescription data from health providers. Using these proprietary data, several recent medication adherence–predictive models were developed and deployed. For instance, Express Scripts developed a model with 300 predictors [5]. These predictors include the patient’s demographic as well as clinical and genomic indicators. The Express Scripts model was reported to have a prediction accuracy of over 90% with a lead time of 6-12 months. Similar proprietary models were also developed by Allazo Health and FICO [6,7].

These models, although successful, primarily rely on proprietary data accessible to the health provider. These data tend to be structured and relatively accurate, providing models with high predictive accuracy [5]. However, the proprietary nature of both the data and the predictive models hinders their widespread use by other health service providers. Due to these limitations, several research efforts started to explore the use of data from social media for large-scale analysis of trends in population health. For instance, social media data were used to build a machine learning model that can predict stress [8]. Twitter data were used to study allergen effects and monitor adverse events of pharmaceutical products across the United States [9,10]. Social media was also used as a mechanism for engaging patients in order to improve compliance [11,12] and assist nonadherent patients [13]. Recently, a medication-adherence model using Twitter was proposed by Klein et al [14]. This model identifies the medication intake from tweets that mention at least one of 55 different medications.

The abovementioned studies highlight the fact that medication-adherence models, in particular, and population health models, in general, have been progressing along parallel but completely disjointed paths. The first path draws its advantage from the accuracy and validity of the data collected in a controlled environment at the expense of limited applicability to the wider population, whereas the second path leverages widespread accessibility, but suffers from reduced data accuracy or lack of verifiable model validity. The objective of this paper is to answer the question: *Can machine learning models trained using data from a controlled environment be used to predict medication adherence for health social forum users?* If they can be used, this approach can bridge the abovementioned parallel paths and help combine the benefits of the two environments.

In machine learning, transfer learning is used to improve modeling in various domains including social media data [15,16,17]. This technique is similar to the ability of a human to transfer knowledge from one context to another, thereby reducing the learning efforts required with every new context. This approach has not been previously explored for medication adherence. The aim of this paper is to investigate the applicability of this approach to medication adherence among patients with fibromyalgia. Fibromyalgia was selected to demonstrate the proposed approach because of its high incidence rate and the fact that it is subject to strict medication regimens with severe consequences of nonadherence [18]. The proposed medication-adherence models for this disease are trained using the Medical Expenditure Panel Survey (MEPS) dataset [19]. Although it is not proprietary, the MEPS dataset is used in this paper as a proxy for datasets collected in a controlled environment. The target domain for knowledge transfer is the social health forum PatientsLikeMe [20]. This paper investigates the accuracy of MEPS-trained models when used to predict the adherence of PatientsLikeMe users in the case of fibromyalgia. The mapping between the variables in the source and target datasets and its impact on the prediction accuracy of the proposed models are also analyzed.

## Methods

### The Machine Learning Model

Typically, a machine learning model is an agent trained with a set of predictors to generate a target outcome. This model varies based on the dataset used for the training and validation as well as the technique used to train each model. Moreover, traditionally, each model is trained and validated using data from a single source, since the learning and application are confined to a single domain. In this paper, and because we are learning from one domain and applying this knowledge to a different domain, two datasets are needed. These datasets are derived from MEPS and PatientsLikeMe.

### Data Extraction and Cross-Domain Variable Mapping

The MEPS database is provided by the Agency for Healthcare Research and Quality [19]. It is a collection of surveys from a nationally representative population of individuals. The survey participants provide responses in a series of five rounds over a 2-year interval. During each round, participants are asked to answer a survey questionnaire that focuses on their health status, medical conditions, prescribed medications, and insurance coverage. Each year, a new panel of participants is enrolled in the study, while the previous year’s panel finishes the final second year. This panel overlap provides an insight into nationwide dynamic changes. For the purpose of this study, patient records were extracted from panels 17-19, which span the period from 2012 to 2015.

The second data source is PatientsLikeMe, which is a social health forum where patients post, discuss, and review many of their current medications and conditions [20]. Some of the users of this forum make their data publicly available. The data were collected from treatment evaluations as of March 2017. These evaluations are available in a structured format that includes self-reported adherence to treatment.

Patients from MEPS and PatientsLikeMe were selected if they were receiving a treatment associated with the target disease fibromyalgia. Treatments were included in the list if they were taken by at least a single patient from PatientsLikeMe. This list includes duloxetine, gabapentin, pregabalin, tramadol, and zolpidem.

The model predictors that were extracted for each patient from both data sources are type of medication, years taking treatment, daily intake, dosage, age at the end of the study or last known age, sex of the patient, out-of-pocket expense, and region of living of the patient (ie, Northeast, Midwest, West, or South). These were the only predictors available in both MEPS and PatientsLikeMe.

As previously mentioned, typical machine learning models rely on a dataset from a single domain for training and validation. However, because we want to transfer knowledge from one domain to another, mapping is needed from the variables in the source dataset to their counterpart in the target dataset. This mapping is straightforward (ie, one to one) in the case of the first six predictors (ie, type of medication, years taking treatment, daily intake, dosage, age, and sex).

However, more elaborate mapping was needed for out-of-pocket expense and region of living. Although MEPS provides the exact amount paid for each medication, PatientsLikeMe lists only ranges for the approximate expenses each month. Therefore, out-of-pocket expense payments in MEPS were categorized using the out-of-pocket expense ranges provided in PatientsLikeMe (Table 1). Similarly, the residence of each MEPS patient is provided according to the appropriate US census region, while for PatientsLikeMe, the residence of the patient is provided at the state level. Again, since one-to-one mapping between the two datasets is needed for knowledge transfer, the value of region of living for PatientsLikeMe was mapped to the census region based on the state of residence of the patient (eg, Indiana is mapped to the Midwest).

**Table 1:** Categorical ranges for out-of-pocket expenses.

Actual expenses (US $)	Out-of-pocket expense category
<25	0
25-50	1
50-100	2
100	3
>200	4

The mapping for the out-of-pocket expense and region of living predictors between the two datasets is relatively simple. The challenge is mapping the outcome of the model. The target outcome for the model is medication adherence. In PatientsLikeMe, patients self-report a selected adherence value from four categories (ie, *Always taken as prescribed*, *Usually taken as prescribed*, *Sometimes taken as prescribed*, or *Never taken as prescribed*). The MEPS data do not include a direct measure of adherence; therefore, this measure had to be derived. In a previous study, Hess et al evaluated 11 different medication-adherence metrics and recommended the use of medication-refill adherence (MRA), which is defined as the total number of days of medication supply divided by the number of days of study participation multiplied by 100 [21]. For example, a patient with a total of 200 days of supply over a period of 365 days will have an MRA of 55%.

The MRA value from MEPS and the adherence classes from PatientsLikeMe have to be mapped to a common scale. This scale consists of two classes: adherent and nonadherent. For PatientsLikeMe, the *Always taken as prescribed* category is mapped to the adherent class and the remaining three categories (ie, *Usually taken as prescribed*, *Sometimes taken as prescribed*, and *Never taken as prescribed*) are mapped to the nonadherent class. In the case of MEPS, four different MRA thresholds are considered. For each threshold, if the MRA is greater than or equal to the threshold value, the outcome is mapped to the adherent class; otherwise, the outcome is mapped to the nonadherent class. The threshold is varied in order to understand the differences in the interpretation of adherence between the MEPS and the PatientsLikeMe datasets. This difference can be due to the fact that adherence is quantitative in MEPS and qualitative in PatientsLikeMe. Moreover, using MRA as an adherence measure in MEPS does not account for scenarios where patients are proactive in refilling their prescriptions or accidentally misplace medications. Finally, adherence in PatientsLikeMe is self-reported and may therefore be subjective [22]. Understanding the differences between the variables in the two datasets and calibrating the associated mapping is a necessary enabler for transfer learning.

### Model Training and Validation

The model proposed for prediction of medication adherence is based on the random forest (RF) tool [23]. Other machine learning techniques (eg, neural networks and support vector machine) are available [24,25]. Although models based on these techniques can be considered for medication adherence, RF was selected for this study because (1) it can handle variables with missing and categorical values, a characteristic inherent to social forum data [23]; (2) it facilitates the comparative analysis of two models trained by using different datasets including the evaluation of the importance of each predictor in each model [26], which is needed for the validation of transfer learning; and (3) it was successfully used in previous health-related models including models to predict the response of patients to various drugs and models to predict patients with liver disease [27,28]. Previous studies [27,28] showed that RF outperformed other machine learning techniques including neural networks and support vector machine.

RF consists of an ensemble of decision trees, where each tree contributes a vote to the overall decision of the RF. A majority vote of adherent or nonadherent classifies the patient as adherent or nonadherent, respectively. The uniqueness of each tree is ensured through a two-step randomization process. The first step is called bagging or bootstrap aggregation and is responsible for randomizing the patients [29]. For each tree in the RF, a predefined number of patients are selected randomly from the training dataset with replacement. Based on this selection, a given patient may be selected more than once in a given tree, while other patients may not be selected. The second randomization step selects predictors and occurs during the construction of each tree. Only a random subset of the predictors is considered at each node. In this paper, the size of this subset was set to a typical standard of Standalone Equation 1, where *n* is the total number of predictors in the dataset. Randomized selection of both patients and predictors from the training dataset helps generate multiple unique decision trees in the RF ensemble.

The best predictor among the Standalone Equation 1 predictors considered at each node of the decision tree is selected based on the greatest reduction in impurity [30]. The parent node in the tree always has a higher impurity (less homogenous set of patients) than its children. A homogenous set of patients corresponds to the case where all the patients belong to the same class (ie, adherent class or nonadherent class). The impurity of a node is defined by *I*=1-(A_+_)^2^–(A_–_)^2^, where A_+_ and A_–_ are the percentage of adherent and nonadherent patients, respectively, presented to the node in a given tree [30]. The change in impurity between the parent node (*p*) and its left (*l*) and right (*r*) child nodes is given by *Δ* *I*= *I*_*p*_– *P*_*l*_*I*_*l*_– *P*_*r*_*I*_*r*_, where *P*_*l*_ and *P*_*r*_ represent the percent of the total number of patients in the parent node that are mapped to the left and right branches, respectively.

In order to split the patients into the appropriate right or left branches at each node, the selected predictor requires a reference value. All possible values for a given predictor are iteratively evaluated until the best split is found (ie, branches with the lowest impurity). In general, predictors can be numeric or categorical. For instance, the predictor age is numeric. When age is used as a predictor for a given node in the tree, patients with an age value greater than the reference value are assigned to the right branch of the node, and the remaining patients are assigned to the left branch. For the categorical predictors, such as region of living, patients that have the same value as the reference value are assigned to the right branch, while the remaining patients are assigned to the left branch.

The abovementioned procedure describes the traditional learning process used for decision trees [23]. One of the limitations of this process is that it does not adequately handle patients with missing predictor values. As previously mentioned, missing predictor values are prevalent in social media data. Specifically, in the PatientsLikeMe dataset, 40% of the patients with fibromyalgia had at least one missing predictor value. Several approaches can be considered to handle missing predictor values: (1) A default split can be adopted, where the patient with a missing predictor value is arbitrarily yet consistently assigned to a given branch. (2) The corresponding patient record can be removed. (3) All numeric predictors are binned using k-means clustering [27]. An additional bin is then added to represent the case where the predictor value is missing.

All of the abovementioned approaches were investigated and discarded because of their limitations. The default split is arbitrary and leads to poor accuracy. The second approach excludes approximately half of the patients from PatientsLikeMe. The third approach translates all numerical values to categorical values. In order to overcome the limitations of these previous approaches, a new technique for learning with missing values in RF models is proposed.

Traditional RF models use decision trees that are binary trees, where each node has one left and one right child. The proposed model uses ternary decisions trees, where each node has three children: a left child, a middle child, and a right child. Patients with missing predictor values are assigned to the third child. The underlying learning algorithm is modified in order to accommodate the additional child and to ensure that the missing value is never selected as a reference in the split at any node.

RF models for fibromyalgia are trained using the abovementioned approach. The training dataset is composed of patient records that include the predictors and the target outcome (ie, adherent/nonadherent). It is also balanced and consists of an equal number of adherent/nonadherent patients. This class balance eliminates the potential of bias in the model toward the larger represented class. The model is then validated using a testing dataset that is completely independent from the training dataset. Two metrics are used to quantify this validation: (1) Accuracy: The ratio of the number of records that are correctly predicted to the total number of records in the testing dataset and (2) F_1_ score: A composite metric that represents a weighted balance between the recall and the precision of the models. Recall accounts for the number of correctly classified adherent patients compared with the total number of actually adherent patients in the testing dataset. Precision is the total number of correctly classified adherent patients against the total number of patients classified as adherent.

Another measure is used to evaluate the importance of each predictor in the model. Predictor importance (PI) is defined as the ratio of the number of times a predictor is traversed to the total number of times all the predictors are traversed in the RF model when processing the testing dataset. As described earlier, the decision trees in the RF model are built by selecting predictors that provide the greatest reduction in impurity. Although there are measures to ensure that the same predictor is not selected repeatedly at each node, the number of times a predictor is selected is indicative of its relative entropy compared to other predictors. Therefore, the higher the PI, the more important the predictor is in the model.

### Transfer Learning

In an ideal case, a model trained on MEPS patients should be able to predict medication adherence for patients from PatientsLikeMe with the same level of accuracy. As an analogy, a human trained to drive a given vehicle should be able to transfer this knowledge to the driving of a different vehicle. However, transferring this knowledge is not simple in either case. The success of this transfer is subject to disparities in data-collection methods, variable mapping, and population distribution between the source and target domains.

Despite the abovementioned difficulties, transfer learning offers several benefits. For instance, the approach has been used to transfer user behavior and content knowledge from one social network to another [15,16]. The approach has also been used for the predictive modeling of the relationship between transcription factor-binding sites and gene expression from one human cell line to another [17]. Transfer learning can help relax the accuracy requirements that are often associated with traditional machine learning methods [31]. Specifically, it can make a medication-adherence model that is developed and validated in a controlled environment accessible to a large-size population with a limited financial burden through, for example, social health forum services. In general, the proposed approach exemplifies the transfer of health models from the proprietary domain to the public domain.

Working toward this objective, a medication-adherence model was initially trained on MEPS patients using all the predictors extracted from the dataset. It was then tested on both the MEPS and the PatientsLikeMe patients. Ideally, the model should be able to predict medication adherence for both population groups with the same level of accuracy. However, this transfer was dependent on the adequacy of the mapping between the predictors and the outcomes in the two domains. This aspect is particularly important in this study owing to the absence of an absolute ground truth for medication adherence and the fact that adherence is equated to medication refills in MEPS and to a subjective self-reported assessment by the patient in PatientsLikeMe.

In order to understand the potential and limitation of transfer learning for medication adherence between MEPS and PatientsLikeMe, the following procedure was adopted:

- The prediction accuracy results for both domains were compared. A significant difference in the accuracy between the two domains is indicative of predictors that fail to transfer from the source domain (MEPS) to the target domain (PatientsLikeMe).
- A secondary model was developed by using the target domain dataset (in this case, PatientsLikeMe). The purpose of this model is to provide an understanding of the differences in the importance of each predictor across the two domains.
- Guided by the abovementioned analysis and using a reductionist approach, a set of predictors that do not transfer from MEPS to PatientsLikeMe was removed.
- A revised model was then developed with the remaining set of predictors, which are deemed transferrable between the two domains. The transfer capabilities of the revised model were then re-evaluated using both the MEPS and the PatientsLikeMe datasets.

## Results

### Overview

The findings derived from the application of the methodology described in the previous section are presented below. These results highlight the salient characteristics of the datasets, the predictive accuracy of the model developed by using the traditional machine learning approach, and the potential applicability of this model to a different domain when appropriate transfer learning requirements are taken into consideration.

### Data Extraction and Cross-Domain Variable Mapping

The demographic breakdown of the MEPS and PatientsLikeMe cohorts is shown in Table 2. The average PatientsLikeMe patient was about 10 years younger than the average MEPS patient. With respect to the region of residence, the largest difference was observed in the southern region. The MEPS patient population in this region accounted for approximately 40% of the total population. However, the PatientsLikeMe dataset had a southern patient population that barely exceeded 30%. Moreover, compared to male patients, female patients accounted for the majority of the cohort for both datasets. However, the female population was significantly larger in the PatientsLikeMe (91%) dataset compared to the MEPS (63.9%) dataset.

In addition to understanding the differences in the demographic distribution of the patients across the two domains, an understanding of the distribution of the patients into adherent/nonadherent classes is crucial. As previously mentioned, the MEPS dataset does not contain a direct adherence metric. Therefore, based on previous studies [21], adherence was derived from the MRA. Since the MRA threshold that distinguishes between adherent/nonadherent patients is not known a priori, several values (ie, 35%, 45%, 65%, and 80%) for the threshold were considered. The distribution of the adherent/nonadherent MEPS patients for each threshold is shown in Table 3. PatientsLikeMe patients self-reported adherence (adherent: n=281 [79%]; nonadherent: n=76 [21%]).

**Table 2:** Demographics of MEPS^a^ and PatientsLikeMe patients with fibromyalgia.

Characteristic		MEPS patients (N=3044)	PatientsLikeMe patients (N=357)
Age (years), mean (SD)		58.0 (14.9)	49.1 (10.7)
**Sex, n (%)**			
	Male	995 (32.7)	27 (7.6)
	Female	1945 (63.9)	325 (91.0)
	Unknown	103 (3.4)	5 (1.4)
**Region, n (%)**			
	Northeast	399 (13.1)	55 (15.4)
	Midwest	651 (21.4)	84 (23.5)
	South	1309 (43.0)	110 (30.8)
	West	560 (18.4)	92 (25.8)
	Unknown	125 (4.1)	16 (4.5)

^a^MEPS: Medical Expenditure Panel Survey

**Table 3:** Distribution of adherent and nonadherent Medical Expenditure Panel Survey patients for varying medication refill–adherence thresholds.

MRA^a^ threshold (%)	Adherent patients, n (%)	Nonadherent patients, n (%)
80	714 (23)	2330 (77)
65	906 (30)	2138 (70)
45	1314 (43)	1730 (57)
35	1571 (52)	1473 (48)

^a^MRA: medication-refill adherence.

### Model Training and Validation

Models for fibromyalgia were trained and tested using the MEPS dataset at different MRA threshold values. For each model, the dataset was split into a training and a testing dataset following an 80/20 split. Moreover, patients were randomly removed from the higher represented class in each training dataset until a 50/50 balance between adherent and nonadherent patients was obtained. For instance, 1616 nonadherent patients selected at random were removed from the model at the 80% MRA threshold.

The results in Table 4 show that the highest predictive accuracy was obtained when the MRA threshold was set to 35%. This indicates that the MEPS models are better at differentiating between extremely nonadherent patients and moderately or highly adherent patients. Based on this result, the MRA threshold of 35% was adopted in the remainder of the study. An intended direction for future work is investigating multiclass adherence models that can differentiate between highly and moderately adherent patients.

### Transfer Learning

The MEPS fibromyalgia model developed in the previous section was tested using the PatientsLikeMe dataset. The prediction accuracy for this dataset was 54.9%, which is significantly lower than the accuracy obtained with the MEPS dataset (76.2%; Table 4). This significant difference indicates that some of the predictors did not adequately transfer from MEPS to PatientsLikeMe. To investigate this result, the PI values for the predictors in the MEPS model were compared to the PI values for the predictors of a secondary model that was trained using the PatientsLikeMe dataset (Table 5).

Predictors with large differences in PI values across the two domains suggest that a predictor has a higher significance in one domain than in the other domain. Based on the results of Table 5, the PI values of both daily intake and out-of-pocket expense differ by approximately 4% across the two domains, while none of the other predictors show a difference of more than ~1%.

Guided by this result, a reduced MEPS model was created after the elimination of the two predictors daily intake and out-of-pocket expense. The performance of the reduced model for both testing datasets (ie, MEPS and PatientsLikeMe) is reported in Table 6. Removing the two predictors significantly improved the accuracy and the F_1_ score for the PatientsLikeMe dataset. As expected, however, for the MEPS testing dataset, a slight reduction in accuracy was observed in the reduced model as compared to the original model. Typically, in traditional machine learning methods, more predictors yield higher accuracy models. However, for transfer learning, these predictors must also map adequately from the source domain to the target domain.

**Table 4:** Accuracy and F_1_ scores of fibromyalgia medication-prediction models that were trained and tested using the Medical Expenditure Panel Survey dataset. Models were created for varying medication refill–adherence thresholds.

MRA^a^ threshold (%)	Patients in the training dataset, n (%)	Patients in the testing dataset, n (%)	Model accuracy (%)	F_1_ score
80	1143 (80)	285 (20)	70.2	72.5
65	1450 (80)	362 (20)	69.9	70.0
45	2103 (80)	525 (20)	73.0	74.6
35	2436 (80)	609 (20)	76.2	77.1

^a^MRA: medication-refill adherence.

**Table 5:** Predictor importance for each predictor in the MEPS^a^–trained model and the PatientsLikeMe-trained model. Both models were tested using the MEPS dataset.

Predictor	Predictor importance	
	Model trained by MEPS	Model trained by PatientsLikeMe
Type of medication	24.2	24.7
Years taking treatment	11.9	12.5
Daily intake	11.8	5.3
Dosage	14.4	14.4
Out-of-pocket expense	9.7	13.6
Region of living of the patient	8.5	9.6
Age at the end of the study or last known age	15.2	16.1
Sex of the patient	4.3	3.8

^a^MEPS: Medical Expenditure Panel Survey.

**Table 6:** Accuracy and F_1_ score of the reduced MEPS^a^–trained model for the MEPS and PatientsLikeMe testing datasets. For comparison, the second row category of the table repeats the results previously obtained for the original model with all predictors from Table 4.

Model		MEPS dataset	PatientsLikeMe dataset
**Reduced model (without daily intake and out-of-pocket expense)**			
	Accuracy (%)	73.2	67.8
	F_1_ score	73.9	79.7
**Original model (with daily intake and >out-of-pocket expense)**			
	Accuracy (%)	76.2	54.9
	F_1_ score	77.1	65.5

^a^MEPS: Medical Expenditure Panel Survey.

Additional investigation is needed to analyze why some of the predictors transfer, whereas others do not. We speculate that some of the root causes can be attributed to potential over/underreporting by the patients [22] and to differences in the sociodemographic distribution of the patients. For instance, 80% of the MEPS patients had an out-of-pocket expense <US $25 each month, whereas less than 40% of the patients in PatientsLikeMe reported an out-of-pocket expense <US $25.

## Discussion

### Principal Results

This study shows that it is possible to develop and validate a model for fibromyalgia medication adherence in a controlled environment and then apply it widely through social health forums. A model trained using MEPS patients with fibromyalgia was able to predict adherence with an accuracy of 73.2% and an F_1_ score of 73.9 for other MEPS patients. Traditionally, for this model to benefit a wider population of patients, these patients would have to be enrolled in the MEPS survey, an approach that is neither practical nor cost-effective. This paper shows that the MEPS model can be transferred to the social health forum PatientsLikeMe with careful mapping between the variables in each domain. The proposed approach was tested with PatientsLikeMe patients with fibromyalgia, and the MEPS-trained model was able to predict adherence for these patients with an accuracy of 67.8% and an F_1_ score of 79.7.

### Limitations

An initial design of the model showed that two of the predictors (daily intake and out-of-pocket expense) in MEPS failed to adequately transfer to PatientsLikeMe. Additional investigation is needed to understand the root cause of this lack of transfer. Access to additional demographic information about the patients may help with this investigation. However, information including race, education, and social status was not available in this study. Furthermore, although MRA was previously shown to provide a good estimate of adherence, there are certain cases where this threshold could misclassify a patient as adherent, since there are no assurances that the medication is actually being taken by the patient.

### Future Work

Future work will consider transfer learning in the context of multiclass adherence models (ie, always, usually, sometimes, or never taken as prescribed). In addition, we would like to study transfer learning for other diseases and other datasets including other social media sources. Finally, understanding the impact of missing values, an unavoidable characteristic of social media data, and development of learning techniques that can handle missing values are areas open for continued research.

### Conclusions

The transferability of a model developed and validated in a controlled environment to the wider public provides tremendous possibilities for improved population health. One can imagine a model for medication adherence derived by a health institution deployed in PatientsLikeMe and enabling the users of this forum to receive alerts or targeted educational material when they are flagged to be at risk of nonadherence.

Transfer learning from one domain to another can be extended, perhaps to disease prediction or other health-related models. This research showed that robust models that can systematically transfer from one domain to another are possible and that it is important to understand the limitations of this transfer. We showed that transfer learning between MEPS and PatientsLikeMe produced similar accuracy of medication-adherence prediction. This approach can have a significant advantage, even if this advantage comes at a slight reduction in prediction accuracy compared with costly, institution-specific machine learning models.

## Abbreviations

MEPS: Medical Expenditure Panel Survey
MRA: medication-refill adherence
PI: predictor importance
RF: random forest
